# Biomechanical Factors Predisposing to Knee Injuries in Junior Female Basketball Players

**DOI:** 10.3390/sports12020060

**Published:** 2024-02-16

**Authors:** Néstor Pérez Mallada, María Jesús Martínez Beltrán, María Ana Saenz Nuño, Ana S. F. Ribeiro, Ignacio de Miguel Villa, Carlos Miso Molina, Ana María Echeverri Tabares, Andrés Paramio Santamaría, Hugo Lamas Sánchez

**Affiliations:** 1 San Juan de Dios Foundation, 28015 Madrid, Spain; mjesus.martinez@comillas.edu (M.J.M.B.); 202105644@alu.comillas.edu (A.M.E.T.); 202112462@alu.comillas.edu (A.P.S.); 202118373@alu.comillas.edu (H.L.S.); 2 Health Sciences Department, San Juan de Dios School of Nursing and Physical Therapy, Comillas Pontifical University, 28015 Madrid, Spain; 3 Institute for Research in Technology (IIT), ICAI School of Engineering, Comillas Pontifical University, 28015 Madrid, Spain; msaenz@comillas.edu; 4 Nursing Department, Faculty of Nursing, Physical Therapy and Podiatry, Complutense University of Madrid, 28040 Madrid, Spain; anasofer@ucm.es; 5 Club Deportivo CB Getafe, 28903 Madrid, Spain; ignaciodmv@gmail.com (I.d.M.V.); charlymiso@gmail.com (C.M.M.)

**Keywords:** knee, ACL, isokinetics, biomechanics, basketball

## Abstract

This cross-sectional observational study aims to determine isokinetic normality data at different speeds, and isometric data of ankle and knee joints, in healthy basketball players aged 15–16 years old. The participants were recruited through non-probabilistic convenience sampling. Sociodemographic, anthropometric, and biomechanical variables were collected. The study involved 42 participants. Right-leg dominance was higher in women (85.7%) than in men (78.6%). Men had a higher weight, height, and body mass index compared to women. Statistically significant differences were observed between sex and height (*p* < 0.001). Significant differences were found between sexes in knee flexor and extensor strength at different isokinetic speeds (30°, 120°, and 180°/s), except for the maximum peak strength knee flexion at 180°/s in the right leg. In the ankle, the variables inversion, eversion, and work strength values at different isokinetic speeds and full RoM, by sex, were not significantly different, except for the right (*p* = 0.004) and the left (*p* = 0.035) ankle full RoM. The study found lower knee extensor strength in women, indicating the need to improve knee flexor/extensor strength in women to match that of men, as seen in other joints. The results can guide the development of preventive and therapeutic interventions for lower limb injuries in basketball players.

## 1. Introduction

Basketball, a team sport featuring five-on-five play, involves repetitive offensive and defensive movements. Enduring both high- and medium-intensity periods in the game demands not only good physical condition but also robust musculature and ligaments to cope with jumps and turns [1].

Basketball began as a sport where contact was penalized but has since evolved into an activity in which physical demands are greater and with higher intensity. It has a high technical complexity and tactical variability, and the repetition of movements such as jumps, turns, sudden and abrupt changes of direction, among others, gives rise to injuries in players. It has been observed that the lower extremities are the region with the highest number of injuries in basketball players, mainly at the level of the ankles, knees, and lower back [2,3].

A study on youth sports revealed that the most frequently injured body parts, in descending order, are the ankle (19%), knee (17.3%), and head/face (14.2%). Intriguingly, college players exhibited the highest frequency of ankle injuries during both training sessions and competitions [4]. Sprained ankles were the most common injury in both high school and college, accounting for 22.6% of all player injuries [5].

Basketball injuries, in general, do not seem to show differences between sexes, although women are more prone to injury than men. Epidemiological data at the college basketball level show a total injury ratio (including training and games) of 7.01 injuries per 1000 h of exposure in women and 7.28 in men [6].

Of the knee injuries, the anterior cruciate ligament (ACL) injury is the most common among basketball players. However, the incidence of ACL injuries in women’s basketball exceeds that of men by up to three times [7]. Multiple studies highlight this aspect, not only in basketball, but also in other sports, such as soccer, volleyball, and gymnastics, among others [8], where women have much higher injury rates. According to Herzberg et al. [9], women are at an increased risk of ACL injuries with rates three to six times higher than men. Taylor et al. [10] also observed that up to 16% of female basketball players could suffer an ACL injury over the course of their career, with rates that can be two to four times higher than male athletes.

Women experienced knee injuries in 29% of cases, making it the most common injury, followed by ankle injuries at 22% [11]. However, according to other authors [12], the frequency of the injuries is inverted, with the most frequent injuries being ankle injuries in 19% of cases compared with 17% for the knee. In general, both joints are notable for being the most prone to injury in this female sport. It is important to note that 33% of all injuries were attributed to contact events with another player and 23% were due to non-contact events; ankle sprains are the most common contact injury (43.7%) and ACL tears are the most frequent non-contact injury (67.6%). It was also observed that the pre-season injury rate was higher than injuries during the regular season and post-season. Likewise, a higher proportion of injuries occurred during training (35.7%), compared to injuries during competitions (23.5%) [6]. It is important to note that training is where strength and endurance gains are worked on, with training programs that seek to exhaust the muscle group to produce an adaptation and improved performance [13].

Several causes have been proposed to explain this increased risk of ACL injury amongst females, such as anatomical characteristics of the female knee, hormonal factors, joint laxity, neuromuscular control, and dynamic factors such as jumping and landing biomechanics [10]. Women have also been observed to sustain non-contact ACL injuries when the knee is in flexion between approximately 15° and 27° [14]. All these factors may play a role in the incidence of injury, but there are no normative strength data to indicate gender differences.

Early specialization means that players are confronted with sporting situations and risk factors at an early age. This implies that, earlier and earlier, players must cope with the specific muscular and proprioceptive mechanisms of very technical movements. The neuromuscular system shows a late development with respect to the growth of the extremities, generating a neuromuscular insufficiency, which causes higher loads to be transferred to the knee, which may give rise to an injury [8]. Similarly, it should be noted that the growth rate peaks are also different according to sex, being higher in girls at 12 years of age and at 14 years of age in boys [15,16], an aspect that should be taken into account when analyzing the injury aspects. Finally, in terms of development, another factor is early specialization, which increases the risk of musculoskeletal injury in adolescent players, due to the multiple actions and repeated gestures of regular practice [17,18].

Thus, an assessment of an individual’s muscle capacity is important to identify possible weaknesses related to illness or injury and aging, followed by the prescription and monitoring of an exercise program [19]. In turn, following analysis of isokinetic biomechanical data in soccer and handball, parameters of greater strength were found in handball for women. This information helps us to understand that each sport activity makes different demands on the strength capacity of the players [20]. Thus, the present study has been carried out with the main objective of determining both isokinetic normality and isometric data at different speeds, for the ankle and knee joints of healthy basketball players according to sex, BMI, and dominance, and, as secondary objectives, to analyze whether there are biomechanical differences in the lower limbs between women and men that may be related to functional alterations that trigger knee injuries, and whether there are differences in terms of sex and anthropometric characteristics. Therefore, we hypothesized that women have different strength values to men in knee flexion/extension strength as well as inversion/eversion strength, which predispose them to knee injuries.

## 2. Materials and Methods

### 2.1. Experimental Design

A cross-sectional observational study with convenience sampling was designed. The study was carried out in the Biomechanics Laboratory of the San Juan de Dios’ University School of Nursing and Physical Therapy, Comillas Pontifical University, Madrid, Spain.

### 2.2. Ethical Considerations

This research study had the approval of the Clinical Ethics Committee of the Hospital Clínico San Carlos (Madrid, Spain) (reference C.P.-C.I.15/416-E). The subjects who participated in the study were given a study information sheet, and signed the informed consent form for their participation, which was collected. All information related to the study was treated as strictly confidential and in accordance with European Regulation 2016/679 of 27 April 2016, and the Spanish Ley Orgánica 3/2018, of December 5, on Personal Data Protection and the guarantee of digital rights; the Biomedical Research Law 14/2007, and its 2016 update, had also been considered, guaranteeing the confidentiality and anonymity of data.

### 2.3. Study Subjects

The study included healthy basketball players aged 15–16 years old who belonged to the Cadet Category of the Basketball Federation of Madrid and were born in 2007 and 2006. The following criteria were taken into consideration:

- Inclusion criteria: aged between 15 and 16 years; healthy subjects; being athletes and members of the Community of Madrid; active in sports and authorized to compete.
- Exclusion criteria: any diagnosed systemic affectation; any recent injury; allergy to any of the components of the measurement and/or intervention systems.

All possible contraindications of the evaluation by means of biomechanics equipment, as well as physiotherapy interventions, were framed as exclusion criteria. In turn, all assessment techniques are covered by Law 44/2003 for health professions under the heading of physiotherapy, and are commonly used and recognized by the various professional associations in the Community of Madrid, as well as in the rest of Spain (there is currently no single national professional association).

Sample size was calculated according to Rhyu et al.’s [21] data, accepting an alpha risk of 0.05 and a beta risk of 0.2 in a bilateral contrast. Forty subjects were required to detect a difference equal to or greater than 9.13 units. A standard deviation of 18.83 and a loss-to-follow-up rate of 15% were assumed.

### 2.4. Procedure

All participants were presented with the subject information sheet, and the informed consent form was signed by the subject and the father/mother and/or legal guardian who accompanied the minor during the study.

Data on sex, date of birth, weight, and height were collected.

The following procedure was carried out: after auto-checking the system and confirming the correct operation of the equipment, bilateral measurements were performed through the PRIMUS RS system.

The test was performed first on the right limb and then on the contralateral limb by segments (first the knee and then both ankle joints).

The measurement protocol used the PRIMUS RS 701 tool (BTE Technologies, Hanover, MD, USA) [22,23], based on previous studies [24,25], where it is recommended to perform at least three different speeds between 30°–240°/s, and an increase of repetitions [26] as the evaluation speed increases. The isokinetic movements of each test (constant speeds) were performed continuously and without rest between flexion/extension or inversion/eversion movements. Between each test, at different speeds, there was a 1 min rest. At the isometric test level, 6 s of contraction with 12 s of rest were performed after each maximal effort. Once the group of three repetitions was finished, 1 min of rest was maintained.

Once the measurements had been collected by the dynamometer and after following the protocol, the data were recorded in an Excel table to export the parameters of each patient to the SPSS data analysis software (version 26.0, IBM Corp. Armonk, NY, USA). These data were extracted numerically by two researchers.

Knee (bilateral onset in dominant). Lever arm 30 cm.

**Figure 1:**
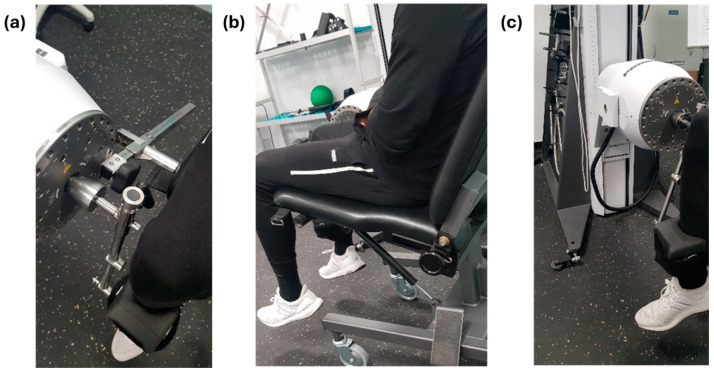
Photographic record of (**a**) position of the lever arm and the chair attachment to the dynamometer axis; (**b**) backrest of the chair; (**c**) lever arm and height of the dynamometer with respect to the floor.

1. Isokinetic flexion/extension, concentric–concentric mode: support on the ventral aspect of the ankle (Figure 1).

Speeds: (i) 30°/s × three repetitions; (ii) 120°/s × five repetitions; (iii) 180°/s × ten repetitions.

Ankle (bilateral onset in dominant). Lever arm 17 cm.

1. Isokinetic inversion/eversion, concentric–concentric mode: knee at 45° flexion/extension.

Speeds: (i) 30°/s × three repetitions; (ii) 90°/s × five repetitions; (iii) 120°/s × ten repetitions.

**Figure 2:**
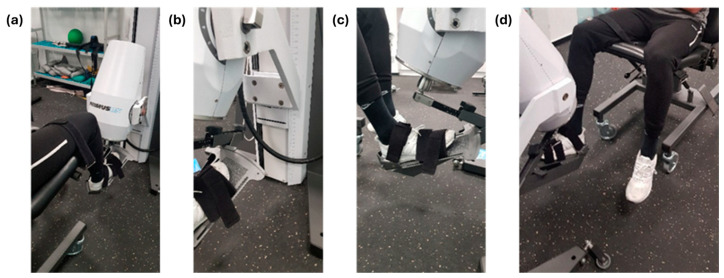
Photographic record of positioning: (**a**) orientation in linear arrangement of the ankle on the dynamometer; (**b**) height of the dynamometer with respect to the ground for the ankle; (**c**) placement of the ankle at 90° on the axis of the dynamometer on the tibiofibular mortise; (**d**) 90° flexion of the knee and ankle with respect to the dynamometer.

2. Isometric inversion and eversion in anatomical ankle position (more details in Figure 2).

### 2.5. Data Collection and Analysis

The data were collected through a data collection table in Microsoft Excel^®^, (version 18.0) encompassing all the variables in our study. Subsequently, the data were transferred to the software IBM SPSS Statistics (version 26.0, IBM Corp. Armonk, NY, USA) for comprehensive analysis. In the initial phase of the statistical analysis plan, a detailed descriptive analysis of the quantitative variables was conducted. This involved calculating the mean, standard deviation, median, and quartiles. For qualitative variables, counts and percentages were determined to provide a comprehensive overview of the dataset.

Following the descriptive analysis, inferential statistical analysis was conducted, with a confidence level set at 95%. Normality and homogeneity of variances were assessed, utilizing the Kolmogorov–Smirnov and Levene tests. This step ensured the appropriateness of parametric tests. In cases where normality assumptions were met, the hypothesis was tested using a Student’s *t*-test for independent samples, along with Cohen’s *d* for effect size. Conversely, if normality assumptions were violated, the Mann–Whitney U test was employed.

## 3. Results

Forty-two participants took part, of whom 28 were men and 14 were women. Right-leg dominance was 78.6% in men and 85.7% in women.

Weight, height, and body mass index in men were 66 kg (9.27), 1.78 m (0.05), and 20.75 (2.45), respectively, while in women they were 63.46 kg (9.93), 1.67 m (0.07), and 22.34 (2.73), respectively.

Notably, there were significant height differences between sexes (*p* < 0.001), while weight (*p* = 0.436) and BMI (*p* = 0.075) showed no statistically significant variations. So, we found that men were taller, but also heavier, which does not affect BMI. There was a slight trend (not significant) of higher BMI in women than men.

Table 1 presents the values of flexion, extension, and work for both knee movements at different isokinetic speeds, first the right and then the left, by sex; the mean differences between both sexes for all were found to be significant (*p* ≤ 0.05), except for the maximum peak force knee flexion at 180°/s in the right leg (*p* = 0.280).

Table 2 presents the values of the knee hamstrings-to-quadriceps peak torque ratio (H/Q ratio) and the inversion/eversion ratio (I/E ratio) at different isokinetic speeds for both the right and left knees, categorized by sex. There were no differences between sexes in any of these variables. The knee ratios at low speed were below the recommended parameter in both men and women.

Table 3 presents the values of inversion, eversion, and work strengths for both ankle movements at different isokinetic speeds and full RoM, first the right and then the left, by sex, with the mean differences between both sexes for all not being found to be significant (*p* > 0.05), except for the full RoM of the right (*p* = 0.004) and the left (*p* = 0.035) ankle.

Table 4 presents the values of mean strength, peak, and coefficient of variation (CoV) of the inversion and eversion movements are presented, first for the right and then the left, by sex, with the mean differences between both sexes for all being significant (*p*≤0.05), except for the inversion and eversion CoV of both sides.

## 4. Discussion

Through data collection, we found that, in terms of weight, height, and BMI, there were differences between the different sexes by height, but not by weight or BMI, since we found that, although some men were taller, they were also heavier, so BMI was not affected.

Significant differences were found between sexes in the lower isokinetic strength of flexion and extension of the knee joint in almost all movements. In the ankle, however, the values of isokinetic strength between sexes were not significant, except in the isometric movements. The variables of H/Q and I/E ratios were similar between sexes.

No differences were observed between sex and BMI, which agrees with data from studies with children up to 12 years old [27] and with adults [28]. The data were within the mean percentiles for the age of the sample [29], being 20.75 (2.45) kg/m^2^ in men and 22.34 (2.73) kg/m^2^ in women. Having a different BMI implies needing different strengths to be able to develop the movements by having to move different masses with respect to their heights. In turn, high BMIs tend to hinder recovery in knee injuries [30], which is also related to a greater number of alterations in the lower limb [31]; in this case, having a similar sample with respect to sex, without significant differences with respect to BMI, should provide similar strength parameters in all muscle groups.

The strength and work at different isokinetic speeds for all ankle parameters were identical regardless of the sex of the player. These data coincided with studies carried out in the ankle with isokinetic tests similar to ours and at similar speeds (30°, 60°, 90°, and 120°/s) where there were no differences in strength in laterality. As shown in Table 1, when comparing the data between sexes for strength and work, we found significant differences in all the measurements with *p* < 0.05 in all cases, except in the variable of strength (Newtons) of the isokinetic test at 180°/s (*p* = 0.280). On the other hand, in the ankle, the differences between sexes were not significant in all cases (*p* > 0.05). The ankle data were equal between sexes, but the knee data were higher in men than in women [32]. This would support the proposition that, at equal BMI, the strength is identical in the full weight-bearing musculature. This aspect was also identical in the relationships between inversion/eversion of the ankle. Regarding the values of RoM of the ankle, women had a greater range of motion which does not correspond to a greater risk of injury [33]. However, in the knee, it should be noted that the RoM was fixed in both sexes at 90° and no free amplitude was left.

It Is known that women are at higher risk of ACL injury than men [6]. There are multiple factors that may be the cause of this higher incidence in women [34], one of them being the lack of muscle strength. Regardless of gender, presenting lower strength [35] in the knee musculature in sport [36] increases the risk of injury to the structures of the knee joint complex. In turn, improving strength also reduces the risk of re-injury during injury recovery [37,38]. In this aspect, we found differences in almost all the isokinetic strengths of both quadriceps and hamstrings between sexes, with lower strength in females (significant *p*-values are indicated in Table 2 with an asterisk). When strength is not adequate in the knee, it is known that there would be a greater risk of ACL injury because it cannot stabilize the joint, so it is important to regain strength after injury [39]. Therefore, we can suggest that lower strength in women than in men may be one of the factors favoring an ACL injury. High levels of strength in the knee flexor/extensor musculature can prevent lower limb injuries in athletes, and specifically of the ACL [40], since the strength relationship between the quadriceps and ischiosural musculature is considered a relevant factor in the stability of the knee [41,42,43]. Therefore, an alteration of this musculature may increase the risk of lower limb injuries [20].

The knee H/Q ratios between sexes did not differ statistically, except for those at low speed, which were lower in males than in females. It should be noted that the values of the H/Q ratios in both sexes at low speed were lower than those recommended in the literature [27] as a factor that prevents ACL injuries. These are values for adolescent-aged players, but if the percentage of injuries in players occurs more at this age and in women, we note that protection of the joint is affected by two factors, lower strength and H/Q ratio, where the hamstrings are the weakest. The evidence suggests that strength training at low speeds of hamstrings in both sexes would protect against the risk of injury, especially in females where weakness is much more evident. The question that arises is whether training sessions at these ages include specific work for this musculature and whether they are the same for men and women, which seems unlikely from the data in this study. ACL injury (either by direct or indirect mechanisms) occurs three to six times more frequently in female athletes [44]. Some studies even speak of an incidence up to ten times higher in female athletes [45].

To evaluate the strength ratio of the flexor/extensor musculature, one of the parameters is the hamstrings/quadriceps (H/Q) index [46]. The conventional ratio is between 0.52 and 0.67, and has a positive correlation with the angular velocity of the test. There is also the functional ratio, which has values around 0.79 at low velocities (60°/s) and can exceed the value of 1 at high velocities (240°/s) [46]. It is considered that there is an imbalance of forces, and therefore an increased risk of injury to the lower limb, if the conventional H/Q ratio is less than 0.47 [47].

Continuing with the main objective and corroborating the previous data in the knee, we can see that the isometric ankle test also presented significantly different data in the strength parameters, being once again higher in males for both peak and average strength, with a significance of *p* < 0.05 in all values of inversion and eversion strength in both right and left knees. These contrast with ankle data isokinetic values, where no significance was noted for all values (*p* > 0.05). The assessment was performed at 90° knee flexion, where the work of the calf muscles is diminished by the shortening of the knee RoM. Therefore, the stabilizing musculature of the knee again becomes the ischioperoneus, in which we have already seen that there was a decrease in strength between sexes. Therefore, these data corroborate the previous hypothesis that the weakness of the ischioperoneotibial musculature in females is a very important factor in providing stability in knee control, which is comparatively diminished.

The CoV in the knees for both sexes did not present significant differences, reporting values between 2 and 5 s that did not exceed 6.99 (4.9) in the right ankle in males and 8.47 (7.82) in females. Likewise, in the left ankle, 10.51 (11.02) was observed in males and 7.99 (3.78) in females. In both cases, these values were even lower than the 10% seen in other knee studies [48,49], or even the 15% found in other joints [50]. These values are important because they provide reliability [51] to the collaboration of the subjects.

With the main objective of this study and two important findings being achieved, the data for all the tests performed were presented, broken down by laterality and sex. Given the data are only provided as normative values in a developing population, with implications for future athletes, they cannot be compared to others as these are not available in an isokinetic format at three speeds for knee and ankle, plus an isometric one, neither in adolescents nor adults. There are only studies which compare only the knee, or ankle, or with different speeds and types of movement. Thus, the present data can only give a starting point for preventative and recovery exercises to reduce injuries.

There is a weakness in knee flexor and extensor strength in female basketball players. We know that lower strength parameters for the same proportional load (similar BMI for both sexes) leads to female muscle weakness in comparison to male knee joints. The isometric parameters also indicate greater male strength, which reinforces the idea of a greater stabilizing capacity of the knee for the development of maximum strengths. Since we cannot affect the biomechanical bone structures of the pelvis and knee, nor hormonal factors, the present results indicate that extensor and flexor analytical strength exercises should be performed in the knee to improve this functionality, since it is known that better strength parameters reduce the incidence of knee injuries.

## 5. Limitations

Specific variables of dynamometric tests of strength, work, and CoV in the knee and ankle joints were presented, in a sports population between 15 and 16 years of age who play basketball. This specificity makes it difficult to compare the data of the present study with other studies, since there are no studies that include this specific population group.

Another limitation of the present study is the sample size. However, it could not have been larger because it is a very specific and delimited population group.

## 6. Conclusions

Data were presented on the strength, work, and CoV of the knee and ankle joints in a sports population between 15 and 16 years of age.

Significant differences were found in the variables’ strength and work values between sexes in the knee joint, but not in the ankle joint. Females presented less strength and less flexor and extensor work, in the knee, but not in the ankle. According to the existing literature, women have a higher rate of knee injuries. In turn, a weak musculature in the knee is a risk factor for increasing the injury rate in this joint.

Therefore, the lower strength and work in women’s knees may be a determining factor in this injury situation, although further research is recommended to confirm this.

## Figures and Tables

**Table 1:** Maximum peak strength for flexion, extension, and work strength for both knee movements at different isokinetic speeds, first the right and then the left, by sex.

	Sex	N	Mean	SD ^1^	Median	Q1–Q3 ^4^	Mean Difference between Sexes	Sig. (Bilateral)	Cohen’s *d*
Right knee—flexion peak strength ^2^ at 30°/s	Men	28	191.25	34.27	185.00	168.00–217.00	20.96	0.030 *	0.665
	Women	14	170.29	24.81	167.00	147.00–189.00			
Right knee—flexion peak strength at 120°/s	Men	28	177.96	48.09	173.00	139.00–209.00	27.10	0.027 *	0.635
	Women	14	150.86	28.28	144.00	133.00–163.00			
Right knee—flexion peak strength at 180°/s	Men	28	187.36	49.87	190.50	148.00–217.00	16.85	0.280	0.359
	Women	14	170.50	40.44	161.00	147.00–191.00			
Left knee—flexion peak strength at 30°/s	Men	28	188.89	33.71	183.50	171.00–204.00	22.32	0.033 *	0.722
	Women	14	166.57	24.14	162.50	152.00–178.00			
Left knee—flexion peak strength at 120°/s	Men	28	181.46	42.16	173.00	153.50–199.50	31.75	0.014 ^a,^*	0.838
	Women	14	149.71	26.84	149.50	125.00–169.00			
Left knee—flexion peak strength at 180°/s	Men	28	194.71	46.51	184.00	162.50–223.50	29.17	0.043 *	0.701
	Women	14 **	165.54	27.53	165.00	145.00–177.00			
Right knee—extension peak strength at 30°/s	Men	28	361.54	75.09	370.00	297.00–431.00	74.03	0.005 *	0.978
	Women	14	287.50	76.86	279.50	215.00–313.00			
Right knee—extension peak strength at 120°/s	Men	28	260.00	71.94	223.50	207.00–331.00	69.07	0.004 ^a,^*	0.998
	Women	14	190.93	63.20	175.00	160.00–229.00			
Right knee—extension peak strength at 180°/s	Men	28	253.32	61.81	245.00	209.50–300.50	46.67	0.027 ^a^*	0.752
	Women	14	206.64	62.69	179.50	165.00–242.00			
Left knee—extension peak strength at 30°/s	Men	28	357.43	64.92	376.50	302.50–411.00	46.92	0.049 *	0.665
	Women	14	310.50	81.13	303.50	265.00–365.00			
Left knee—extension peak strength at 120°/s	Men	28	275.79	65.35	270.00	226.00–329.50	68.14	0.002 *	1.067
	Women	14	207.64	60.56	201.00	160.00–232.00			
Left knee—extension peak strength at 180°/s	Men	28	256.75	56.64	246.00	211.00–311.50	56.28	0.004 *	1.015
	Women	14 **	200.46	52.63	185.00	170.00–237.00			
Right knee—work ^3^ at 30°/s	Men	28	473.18	86.27	475.50	397.50–540.00	69.10	0.014 *	0.844
	Women	14	404.07	71.85	396.50	360.00–483.00			
Right knee—work at 120°/s	Men	28	557.86	143.32	527.00	468.50–656.50	122.50	0.013 *	0.852
	Women	14	435.36	144.86	430.00	340.00–506.00			
Right knee—work at 180°/s	Men	28	1041.79	312.13	1036.50	843.50–1163.00	268.35	0.009 *	0.898
	Women	14	773.43	268.75	714.50	599.00–1082.00			
Left knee—work at 30°/s	Men	28	480.29	82.27	497.50	423.50–541.50	73.00	0.009 *	0.899
	Women	14	407.29	78.89	400.00	368.00–450.00			
Left knee—work at 120°/s	Men	28	616.14	135.44	659.50	532.00–719.50	141.50	0.002 *	1.099
	Women	14	474.64	113.74	457.50	400.00–521.00			
Left knee—work at 180°/s	Men	28	1034.61	289.91	1003.50	804.00–1230.50	219.37	0.019 ^a,^*	0.819
	Women	14 **	815.23	209.88	670.00	670.00–1045.00			

^1^ SD—standard deviation. ^2^ Strength peak (measured in Newtons): this is the maximum value of the repetitions performed. ^3^ Knee work: average strength data for the RoM, taking flexion and extension data (joint) to obtain work data in Joules. ^4^ Q1 is the first quartile data and Q3 is the third quartile data. ^a^ Mann–Whitney U test. For the rest, Student’s *t*-test. * Tests with statistically significant differences for *p* < 0.05. ** The sample size was initially n = 14, but due to the performance of one patient which made the results invalid, the sample was reduced to n = 13.

**Table 2:** Knee H/Q and I/E ratios at different isokinetic speeds, first the right and then the left, by sex.

	Sex	N	Mean	SD ^1^	Median	Q1–Q3 ^3^	Mean Difference between Sexes	Sig. (Bilateral)	Cohen’s *d*
Right knee—H/Q peak strength ^2^ ratio at 30°/s	Men	28	0.5397	0.0920	0.5485	0.4818–0.5804	−0.0791	0.060	0.738
	Women	14	0.6189	0.1336	0.6113	0.5174–0.7131			
Right knee—H/Q peak strength ratio at 120°/s	Men	28	0.7104	0.1950	0.6635	0.5744–0.8158	−0.1481	0.054	0.649
	Women	14	0.8585	0.2851	0.7856	0.6094–1.1167			
Right knee—H/Q peak strength ratio at 180°/s	Men	28	0.7518	0.1471	0.7321	0.6346–0.8850	−0.1001	0.052	0.674
	Women	14	0.8519	0.1516	0.8627	0.7815–0.9388			
Left knee—H/Q peak strength ratio at 30°/s	Men	28	0.5353	0.0805	0.5378	0.4541–0.6048	−0.0265	0.403	0.277
	Women	14	0.5619	0.1224	0.5738	0.4366–0.6284			
Left knee—H/Q peak strength ratio at 120°/s	Men	28	0.6740	0.1372	0.6548	0.5725–0.7473	−0.0910	0.110	0.535
	Women	14	0.7650	0.2235	0.6844	0.5770–0.9522			
Left knee—H/Q peak strength ratio at 180°/s	Men	28	0.7742	0.1720	0.7335	0.6735–0.8526	−0.0817	0.156	0.486
	Women	14 *	0.8559	0.1593	0.8312	0.5770–0.9522			
Right ankle—I/E peak strength ratio at 30°/s	Men	28	1.0479	0.3604	1.0190	0.7802–1.1871	−0.0745	0.493	0.227
	Women	14	1.1225	0.2516	1.0828	1.0013–1.3348			
Right ankle—I/E peak strength ratio at 90°/s	Men	28	1.1420	0.3570	1.0742	0.8414–1.3688	−0.1710	0.209 ^a^	0.410
	Women	14	1.3130	0.5009	1.1821	0.9622–1.6134			
Right ankle—I/E peak strength ratio at 120°/s	Men	28	1.2040	0.4083	1.1215	0.9087–1.5311	0.0135	0.917 ^a^	0.034
	Women	14	1.1905	0.3598	1.1652	0.9890–1.2432			
Left ankle—I/E peak strength ratio at 30°/s	Men	28	1.0256	0.2621	1.0299	0.8383–1.2566	−0.0148	0.880	0.050
	Women	14	1.0404	0.3604	0.9528	0.7299–1.2690			
Left ankle—I/E peak strength ratio at 90°/s	Men	28	1.1216	0.3874	1.0664	0.8421–1.3374	−0.0215	0.878 ^a^	0.050
	Women	14	1.1432	0.4979	1.0163	0.9096–1.2446			
Left ankle—I/E peak strength ratio at 120°/s	Men	28	1.1666	0.2938	1.1515	0.9913–1.3035	−0.0082	0.941	0.024
	Women	14	1.1748	0.4094	1.0512	0.9339–1.3529			

^1^ SD—standard deviation. ^2^ Strength (measured in Newtons): this is the maximum value of the repetitions performed. ^3^ Q1 is the first quartile data and Q3 is the third quartile data. ^a^ Mann–Whitney U test. For the rest, Student’s *t*-test. * The sample size was initially n = 14, but due to the performance of one patient which made the results invalid, the sample was reduced to n = 13.

**Table 3:** Maximum peak of inversion, eversion, and work strengths for both ankle movements at different isokinetic speeds and full RoM, first the right and then the left.

	Sex	N	Mean	SD ^1^	Median	Q1–Q3 ^4^	Mean Difference between Sexes	Sig. (Bilateral)	Cohen’s *d*
Right ankle—inversion peak strength ^2^ at 30°/s	Men	28	129.11	41.50	122.00	96.95–146.50	6.250	0.617	0.165
	Women	14	122.86	28.86	123.50	104.75–138.25			
Right ankle—inversion peak strength at 90°/s	Men	28	117.75	42.87	111.50	85.00–152.00	−9.107	0.544	0.200
	Women	14	126.86	50.31	125.50	83.00–160.00			
Right ankle—inversion peak strength at 120°/s	Men	28	146.96	53.60	136.00	106.25–184.25	−2.321	0.888 ^a^	0.046
	Women	14	149.29	41.41	160.00	132.75–166.50			
Left ankle—inversion peak strength at 30°/s	Men	28	135.07	43.62	124.00	97.25–173.25	23.000	0.094	0.561
	Women	14	112.07	34.81	105.00	91.25–126.50			
Left ankle—inversion peak strength at 90°/s	Men	28	121.11	52.87	105.00	70.75–171.25	2.321	0.891	0.045
	Women	14	118.79	48.08	119.00	68.50–157.25			
Left ankle—inversion peak strength at 120°/s	Men	28	144.75	38.32	148.50	116.25–174.50	10.321	0.391	0.284
	Women	14 **	134.43	31.85	145.50	109.75–160.00			
Right ankle—eversion peak strength at 30°/s	Men	28	128.82	34.81	125.50	100.25–165.75	13.96	0.243	0.388
	Women	14	114.86	38.42	110.50	84.75–140.00			
Right ankle—eversion peak strength at 90°/s	Men	28	107.89	39.55	97.50	79.25–145.50	9.17	0.437 ^a^	0.257
	Women	14	98.71	25.95	100.50	74.75–120.50			
Right ankle—eversion peak strength at 120°/s	Men	28	126.21	35.76	119.00	98.00–151.50	−4.14	0.735	0.111
	Women	14	130.36	39.94	135.00	79.75–162.25			
Left ankle—eversion peak strength at 30°/s	Men	28	134.61	39.33	130.00	106.75–158.75	22.25	0.068	0.614
	Women	14	112.36	28.79	107.50	93.50–124.50			
Left ankle—eversion peak strength at 90°/s	Men	28	110.21	38.35	97.50	78.25–145.75	−0.10	0.994 ^a^	0.003
	Women	14	110.31	38.61	101.00	78.25–140.25			
Left ankle—eversion peak strength at 120°/s	Men	28	129.00	39.32	118.50	100.50–156.25	5.00	0.713	0.121
	Women	14 **	124.00	44.82	117.00	83.25–156.50			
Right ankle—work ^3^ at 30°/s	Men	28	68.93	21.96	62.00	50.00–87.75	3.714	0.598	0.174
	Women	14	65.21	20.00	64.50	45.00–83.50			
Right ankle—work at 90°/s	Men	28	102.86	38.32	104.00	68.25–138.25	9.143	0.469	0.239
	Women	14	93.71	38.02	91.50	59.50–132.75			
Right ankle—work at 120°/s	Men	28	193.29	71.73	203.50	121.50–248.00	11.357	0.628	0.160
	Women	14	181.93	69.50	162.00	126.50–232.00			
Left ankle—work at 30°/s	Men	28	69.64	27.04	66.50	47.00–84.75	7.643	0.365	0.300
	Women	14	62.00	21.87	57.50	45.50–75.25			
Left ankle—work at 90°/s	Men	28	101.71	42.67	93.00	64.75–137.00	16.714	0.209	0.418
	Women	14	85.00	33.85	75.50	60.50–107.75			
Left ankle—work at 120°/s	Men	28	189.46	79.75	168.00	129.25–228.25	28.679	0.240 ^a^	0.391
	Women	14	160.79	57.96	144.00	117.00–215.25			
Right ankle—full RoM inversion/eversion	Men	28	59.64	7.279	59.50	53.50–67.00	−8.929	0.004 *	1.007
	Women	14	68.57	11.48	67.50	59.75–74.50			
Left ankle—full RoM inversion/eversion	Men	28	57.29	7.123	56.00	52.50–60.00	−5.643	0.035 *	0.714
	Women	14	62.93	9.327	60.00	56.75–70.50			

^1^ SD—standard deviation. ^2^ Strength (measured in Newtons): this is the maximum value of the repetitions performed. ^3^ Ankle work: average strength data for the RoM, taking inversion and eversion data (joint) to obtain work data in Joules. ^4^ Q1 is the first quartile data and Q3 is the third quartile data. ^a^ Mann–Whitney U test. For the rest, Student’s *t*-test. * Tests with statistically significant differences for *p* < 0.05. ** The sample size was initially n = 14, but due to the performance of one patient which made the results invalid, the sample was reduced to n = 13.

**Table 4:** Average peak isometric strength, peak strength, and CoV of the inversion and eversion movements, first the right and then the left, by sex.

	Sex	N	Mean	SD ^1^	Median	Q1–Q3 ^5^	Mean Difference between Sexes	Sig. (Bilateral)	Cohen’s *d*
Right ankle—peak strength ^2^ neutral isometric inversion	Men	28	76.55	24.05	75.35	61.10–94.60	20.71	0.005 *	0.982
	Women	14	55.84	12.91	56.30	48.20–63.15			
Right ankle—peak isometric strength^3^ neutral inversion	Men	28	90.66	26.91	88.30	73.32–108.17	20.18	0.013 *	0.964
	Women	14	70.47	14.75	71.90	62.67–79.77			
Right ankle—CoV ^4^ neutral isometric inversion	Men	28	7.98	5.19	7.90	3.20–11.62	−2.01	0.258	0.376
	Women	14	10.00	5.69	8.50	5.77–15.17			
Left ankle—peak strength neutral isometric inversion	Men	28	71.41	30.13	62.00	51.20–84.40	17.17	0.050 *	0.643
	Women	14	54.23	17.63	61.35	34.85–66.67			
Left ankle—peak isometric strength neutral inversion	Men	28	88.14	33.43	80.10	67.97–107.57	22.81	0.022 *	0.778
	Women	14	65.32	18.00	71.55	46.27–76.70			
Left ankle—CoV neutral isometric inversion	Men	28	11.28	7.53	10.35	5.97–14.15	0.84	0.717	0.119
	Women	14 **	10.43	6.04	8.90	6.00–15.52			
Right ankle—peak strength neutral isometric eversion	Men	28	110.62	37.75	108.60	86.40–128.85	25.50	0.025 ^a,^*	0.761
	Women	14	85.12	22.23	90.00	71.07–98.87			
Right ankle—peak isometric strength neutral eversion	Men	28	130.73	39.72	129.00	109.87–144.07	28.61	0.016 *	0.822
	Women	14	102.12	21.25	109.60	83.67–115.52			
Right ankle—CoV neutral isometric eversion	Men	28	6.99	4.91	6.10	3.30–9.15	−1.47	0.457	0.246
	Women	14	8.47	7.82	7.60	3.97–9.32			
Left ankle—peak strength neutral isometric eversion	Men	28	104.66	34.16	97.25	80.67–120.92	30.15	0.003 *	1.017
	Women	14	74.50	16.74	75.15	63.52–83.17			
Left ankle—peak isometric strength neutral eversion	Men	28	127.23	37.13	120.95	99.22–148.07	36.95	0.001 *	1.175
	Women	14	90.28	13.43	87.75	81.05–99.45			
Left ankle—CoV neutral isometric eversion	Men	28	10.51	11.02	7.85	5.02–11.52	2.51	0.414 ^a^	0.270
	Women	14	7.99	3.79	7.75	4.75–9.25			

^1^ SD—standard deviation. ^2^ Maximum average strength (measured in Newtons). Of the three repetitions of maximum isometric strength lasting 6 s, the average of the values between 2 and 5 s was obtained. ^3^ Peak strength (measured in Newtons). Of the three repetitions of maximal isometric strength lasting 6 s, the highest peak value was selected. ^4^ CoV—coefficient of variation (SD/average) × 100. ^5^ Q1 is the first quartile data and Q3 is the third quartile data. ^a^ Mann–Whitney U test. For the rest, Student’s *t*-test. * Tests with statistically significant differences for *p* < 0.05. ** The sample size was initially n = 14, but due to the performance of one patient which made the results invalid, the sample was reduced to n = 13.

## Data Availability

The data presented in this study are available in the article.
